# Sex differences in the impact of ventricular-arterial coupling on left ventricular function in patients with hypertension

**DOI:** 10.1371/journal.pone.0313677

**Published:** 2024-11-19

**Authors:** Seung-Jae Joo, Ki Yung Boo, Jae-Geun Lee, Joon-Hyouk Choi, Song-Yi Kim

**Affiliations:** 1 Department of Internal Medicine, Jeju National University Hospital, Jeju, Republic of Korea; 2 Department of Internal Medicine, Jeju National University College of Medicine, Jeju, Republic of Korea; University of Southern California, UNITED STATES OF AMERICA

## Abstract

Increased arterial stiffness elevates aortic load, which can have adverse impacts on left ventricular (LV) function and contribute to the onset of heart failure. This impact is known to be more pronounced in women. Optimal coordination between ventricular contraction and the arterial system is required to maintain efficient cardiac function. This study aimed to investigate sex differences in the impact of ventricular-arterial coupling (VAC) on LV function in patients with hypertension at rest and after handgrip exercise. Echocardiographic indexes of LV volumes, systolic function, and diastolic function were obtained in the usual way. Effective arterial elastance (E_A_) and index (E_A_I) were calculated from stroke volume measured using LV outflow waveform. Effective LV end-systolic elastance (E_LV_) and index (E_LV_I) were obtained using the single-beat method. Central aortic pressure waveform was recorded using the applanation tonometry. Characteristic impedance (Zc) of aortic root and reflection magnitude (RM) was calculated after Fourier transformation of both aortic pressure and flow waveforms. Sixty-four patients (31 women and 33 men) with hypertension were enrolled. Women showed higher E_LV_I (1.33±0.34 vs. 1.10±0.29 mmHg/ml∙m^2^, *P* = 0.004) and E_A_I (1.14±0.25 vs. 0.93±0.26 mmHg/ml∙m^2^, *P* = 0.001), but VAC was not different (women: 0.88±0.17 vs. men: 0.85±0.11, *P* = 0.431). Zc and RM were not different between women and men. After handgrip exercise, an increase in E_LV_I (*P* = 0.021) and a decrease in VAC (*P* = 0.035) were observed specifically in men, with no corresponding changes noted in women. In women, VAC was significantly associated with E’ velocity (beta -0.344, *P* = 0.029) and left ventricular global longitudinal strain (beta 0.470, *P* = 0.012) after adjustment, but in men, no association was found. Hypertensive women demonstrated greater stiffness in both the left ventricle and arterial systems, along with impaired LV contractile reserve in response to handgrip exercise, as compared to men. The ventricular-arterial mismatch had a notable impact on LV diastolic and systolic dysfunction only in women, but not in men.

## Introduction

The prevalence of heart failure (HF) is increasing globally, and it is thought to affect 1% to 2% of all adults in developed countries [1]. HF continues to be a major public health concern with a high economic burden due to the increasing number of patients, considering its chronic nature and the aging population. HF is usually classified using left ventricular (LV) ejection fraction (EF) as a phenotypic marker: HF with reduced EF (HFrEF; EF <40%), mildly reduced EF (HFmrEF; EF 40–49%), or preserved EF (HFpEF; EF ≥50%) [2]. Among these phenotypes, HFpEF is characterized by a high prevalence in the elderly, women and individuals with arterial hypertension [3], all of whom have increased arterial stiffness.

Both forward and reflected wave velocities increase with rising arterial stiffness. As a result, there is an increase in aortic systolic and pulse pressures, and a reduction in diastolic pressure due to the overlapping reflection wave during mid-systole. The elevated aortic systolic pressure increases myocardial oxygen demand and LV afterload. Concurrently, the decreased diastolic pressure in the aorta diminishes blood flow via the coronary arteries, potentially leading to myocardial ischemia. These factors can collectively impair LV function and accelerate the onset of heart failure, particularly HFpEF [4–8]. This impact is known to be more pronounced in women because of women’s shorter stature, smaller large artery diameter, and lower large and small artery elasticity, despite the similar speed of arterial pulsation, pulse wave velocity, in both sexes [9–11]. It has been demonstrated that women exhibit higher aortic stiffness than men throughout their lifespan, particularly in postmenopausal women [11], although the exact mechanisms remain unclear. The decreased direct vascular effects of estrogen are considered an important contributing factor [11]. This increase in aortic stiffness is suggested to play a role in the significant rise in cardiovascular mortality observed in postmenopausal women [12].

In this regard, maintaining efficient cardiac function in women with elevated arterial stiffness necessitates optimal synchronization between ventricular contraction and the arterial system through which blood is circulated. Abnormal interaction between these elements is associated with the development of HF [6, 7]. However, because LV contractile function and arterial load are usually expressed in different units, evaluating their direct connection proved to be difficult. Although intrinsic frequency metrics derived from carotid pressure waveforms have been recently proposed to assess ventricular-arterial coupling (VAC) [13], elastance, which quantifies the rise in pressure relative to volume change, remains frequently used despite its inherent limitations [14, 15]. VAC is measured as the ratio of effective arterial elastance (E_A_) to effective LV end-systolic elastance (E_LV_). While the measurement of VAC and its component can provide incremental insight into the different association of aortic afterload with LV functional change between women and men, it has been the subject of limited investigation. Previous studies have primarily focused on sex differences in the relationships between the ratio of central pulse pressure (PP) to stroke volume (SV) index, brachial-ankle pulse wave velocity or central pressure amplification and LV diastolic dysfunction [16–18]. Consequently, the role of VAC in the context of sex-specific differences in LV diastolic performance remains underexplored.

The impact of VAC on LV function may become more evident with a maneuver that increases the aortic load. An appropriate and practical tool for this purpose is an isometric handgrip exercise, which raises systolic blood pressure (BP) and LV afterload [19–21]. This study aimed to investigate sex differences in the impact of VAC on LV function in patients with hypertension at rest and after handgrip exercise.

## Methods

This study was conducted in accordance with the Declaration of Helsinki. The study protocol received approval from the institutional review board (IRB) at Jeju National University Hospital, Republic of Korea (IRB No. JNUH-2020-12-007). Written informed consents were obtained from participating patients.

### Study patients

Patients with hypertension who were scheduled for a routine echocardiographic study and agreed to participate in the study were enrolled from July 1, 2022 to April 30, 2023. Patients with acute coronary syndrome, stroke within one year, valvular heart diseases, cardiomyopathy, congestive HF, LV EF <50%, those not in sinus rhythm, or with uncontrolled hypertension (systolic BP ≥160 mmHg, or diastolic BP ≥100 mmHg) were excluded. Hypertension was defined as systolic BP ≥140 mmHg or diastolic BP ≥90 mmHg, or when taking antihypertensive medication. Age, gender, height, weight, body surface area (BSA), and co-morbidities such as diabetes mellitus, hyperlipidemia, or angina, as well as past medical history of myocardial infarction, HF, or stroke, smoking status, and medications, were all collected from electronic medical records.

The study protocol is illustrated in S1 Fig, and the lists of the measured and calculated echocardiographic and hemodynamic data are summarized in S1 Table.

### Transthoracic echocardiographic study

The Vivid E95 system (GE Medical, Milwaukee, WI, USA) was employed for transthoracic echocardiographic examinations. Measurements derived from M-mode tracings included LV end-diastolic and end-systolic dimensions (cm), and LV wall thickness (cm) at diastole. Left atrium (LA) end-systolic volume (mL) was determined using the biplane method of discs and indexed by BSA (mL/m^2^). The LV mass index (gram/m^2^) was computed using the Devereux formula [22]. LVEF (%) was calculated based on diastolic and systolic LV volumes (mL), measured using the modified Simpson’s approach from apical 4- and 2-chamber images.

The LV outflow tract (LVOT) area (cm^2^), measured at the parasternal long-axis, and the time-velocity integral (cm) of the LVOT flow obtained by pulsed-wave Doppler echocardiography at the apical 5-chamber view were multiplied to determine SV (mL). Cardiac output (CO, in mL/min) was then calculated using the formula: SV (mL) × Heart rate (HR, in beats/min). The resulting cardiac output was indexed by BSA to obtain cardiac index (CI, in mL/min/m^2^).

Using pulsed and tissue Doppler echocardiographic images, standard diastolic filling parameters were measured at the apical 4-chamber view. These parameters included peak early-diastolic transmitral flow (E wave) velocity (cm/sec), early-diastolic septal mitral annular (E’ wave) velocity (cm/sec), as well as the E/E’ ratio.

From the pulsed-wave Doppler tracing of LVOT flow at the apical 5-chamber view, the ratio of the period from the ECG Q wave to flow-onset to the period from the ECG Q wave to end-flow was obtained. This yielded the ratio of pre-ejection period to total systolic period, referred to as tNd (S2 Fig).

Using software provided by the vendor, LV strain analysis was performed using 2-dimensional speckle tracking images in the apical 2-, 3-, and 4-chamber views. Subsequently, the mean LV global longitudinal strain (GLS, in %) was computed.

### Hemodynamic study

Hemodynamic measurements were taken with the patient in a supine position after a transthoracic echocardiogram. Given that body size significantly impacts hemodynamic parameters [11, 15], we employed BSA as an indexing factor for accurate measurement. A digital sphygmomanometer (Microlife BP A100, Microlife AG, Widnau, Switzerland) was used to measure brachial BP (mmHg). The difference between brachial systolic BP (SBP) and brachial diastolic BP (DBP) was used to calculate brachial PP (mmHg). Adding one-third of brachial PP to brachial DBP yielded the mean brachial BP (mmHg). The mean brachial BP was then multiplied by 80, divided by CO to calculate systemic vascular resistance (SVR, in dynes/sec·cm^-5^), and indexed by BSA (SVRI, in dynes/sec·cm^-7^).

Pressure wave analysis of the pressure waveform at the radial artery, employing applanation tonometry (SphygmoCor^®^, AtCor, Sydney, Australia), was utilized to estimate the central aortic pressure waveform and various parameters. These parameters include aortic augmentation index adjusted to a heart rate of 75 beats per minute (AIx75, in %), central (proximal aortic) SBP, central DBP, central PP, central end-systolic pressure (ESP, in mmHg), and pressure-time indexes (mmHg·sec/min) at systole (sPTI) and diastole (dPTI). The radial pressure waveform was calibrated using brachial SBP and DBP. Total arterial compliance (TAC, in mL/mmHg) was calculated using the following formula and then indexed by BSA to obtain TAC index (TACI, in mL/mmHg·m^2^):

TAC=(dPTI×SV)/[(sPTI+dPTI)×(centralESP−centralDBP)]

[16, 23].

### Measurements of VAC and its components

Brachial SBP multiplied by 0.9 was utilized to estimate ESP. E_A_ (mmHg/mL) was then calculated as ESP divided by SV, and indexed by BSA to obtain E_A_ index (E_A_I in mmHg/mL·m^2^). The single-beat approach was employed to estimate E_LV_ (mmHg/mL), derived from the time-varying elastance curve and tNd obtained from an echocardiographic study [24–26]. It was then indexed by BSA to obtain the E_LV_ index (E_LV_I in mmHg/mL·m^2^). Subsequently, the ratio of E_A_ to E_LV_ was calculated to determine VAC.

### Measurements of aortic characteristic impedance and reflection magnitude

Aortic pressure-flow analysis was conducted using digitalized data of LVOT flow obtained through pulsed-wave Doppler echocardiography and aortic pressure estimated from the radial waveform. The software for aortic pressure-flow analysis was self-programmed using LabVIEW (National Instruments, Austin, TX, USA).

The systolic ejection period was synchronized by aligning the rapid rise in the aortic pressure wave with the initiation of LVOT flow and the dicrotic notch in the aortic pressure with the end of LVOT flow. Following Fourier transformation, the modulus of aortic pressure to LVOT flow in the frequency domain was employed to calculate the aortic input impedance (Zin). The average value of Zin’s third through tenth harmonics was then used to determine the aortic characteristic impedance (Zc). Subsequently, RM was calculated as the ratio of the backward pressure wave (Pb) to the forward pressure wave (Pf) after wave separation analysis using the following formula (S3 Fig):

$$Pf=\left(P+Q\times Zc\right)/2,$$

and

Pb=(P-Q×Zc)/2

Here, P represents pressure and Q represents flow.

### Isometric handgrip exercise

The maximal voluntary forearm contraction power was measured with a JAMAR dynamometer (Sammons Preston Rolyan, Nottinghamshire, UK) before echocardiographic and hemodynamic studies. A submaximal target, set at 30–40% of maximal handgrip power, was utilized for a 2-minute isometric handgrip exercise. After the handgrip exercise, echocardiographic and hemodynamic studies were repeated using the same protocols as those used before the handgrip exercise.

### Statistical analysis

For continuous variables, the data were presented as mean ± standard deviation, and for categorical variables, as a number (%). The normality of the data was assessed using the Kolmogorov-Smirnov test, and the homogeneity of variances was evaluated with Levene’s test. An unpaired t-test was employed for between-group comparisons of continuous data, and the chi-square test was used for categorical variables. The paired t-test was utilized to examine changes in hemodynamic and echocardiographic parameters following the handgrip exercise. Using correlation and linear regression analysis, the relationships between hemodynamic data and E’ velocity, E/E’ ratio, and LV GLS were evaluated, and all statistical assumptions were tested. The statistical software SPSS version 23 (IBM Co., Armonk, NY, US) was used for all statistical analyses. The threshold for clinical significance was set at a *P*-value <0.05.

## Results

### Baseline clinical characteristics

A total of 64 patients (64.7 ± 8.9 years; 31 women, 33 men) were enrolled. Women exhibited smaller height, weight, and BSA, but age and BMI were not different between women and men. The co-morbidities, including DM, hyperlipidemia, angina, MI, HF, stroke or chronic kidney disease, and current smoking status, were not different between sexes. Current medications, including beta-blockers, renin-angiotensin system inhibitors, calcium channel blockers, diuretics, statins, and nitrates, were also not different (Table 1).

**Table 1:** Baseline characteristics of patients.

	Total (n = 64)	Female (n = 31)	Male (n = 33)	*P* value
Age (years)	64.7±8.9	65.7±9.3	63.8±8.5	0.378
Height (cm)	161.5±9.6	154.1±6.8	168.5±5.8	<0.001
Weight (kg)	67.5±11.6	61.7±10.1	72.9±10.3	<0.001
Body surface area (m^2^)	1.71±0.18	1.59±0.14	1.83±0.14	<0.001
Body mass index (kg/m^2^)	25.78±3.31	25.92±3.66	25.65±3.01	0.744
Diabetes mellitus	17 (26.6)	5 (16.1)	12 (36.4)	0.091
Hyperlipidemia	49 (76.6)	21 (67.7)	28 (84.8)	0.143
Prior angina	4 (6.3)	1 (3.2)	3 (9.1)	0.614
Prior myocardial infarction	6 (9.4)	2 (6.5)	4 (12.1)	0.673
Prior heart failure	1 (1.6)	1 (3.2)	0 (0.0)	0.484
Prior stroke	3 (4.7)	2 (6.5)	1 (3.0)	0.607
Smoker	8 (12.5)	1 (3.2)	7 (21.2)	0.054
Chronic kidney disease	3 (4.7)	1 (3.2)	2 (6.1)	1.000
Medications				
Beta-blockers	12 (18.8)	5 (16.1)	7 (21.2)	0.752
ACEi	1 (1.6)	0 (0.0)	1 (3.0)	1.000
ARB	41 (64.1)	19 (61.3)	22 (66.7)	0.795
Calcium channel blocker	37 (57.8)	16 (51.6)	21 (63.6)	0.448
Diuretics	10 (15.6)	5 (16.1)	5 (15.2)	1.000
Statin	40 (62.5)	18 (58.1)	22 (66.7)	0.607
Nitrate	1 (1.6)	1 (3.2)	0 (0.0)	0.484

Values are mean ± standard deviation or number (%).

ACEi, angiotensin-converting enzyme inhibitor; ARB, angiotensin receptor blocker

### Echocardiographic and hemodynamic data

LV diastolic and systolic dimensions, interventricular septum thickness, and LV posterior wall thickness were smaller in women. Additionally, LV diastolic and systolic volumes were also smaller in women. However, LA end-systolic volume index, relative wall thickness, LV mass index, and LVEF showed no significant differences between women and men. SV and CO were lower in women due to a smaller LVOT diameter, but CI was not different. Doppler echocardiographic parameters, including E velocity, E’ velocity, as well as E/E’ ratio, did not show significant differences. LV GLS was lower in women (Table 2).

**Table 2:** Comparison of echocardiographic data.

	Total (n = 64)	Female (n = 31)	Male (n = 33)	*P* value
IVS thickness (cm)	0.95±0.11	0.90±0.10	1.00±0.10	<0.001
LVPW thickness (cm)	0.82±0.10	0.79±0.07	0.85±0.11	0.007
LVIDd (cm)	4.83±0.43	4.70±0.40	4.94±0.42	0.023
LVIDs (cm)	3.00±0.45	2.87±0.43	3.12±0.45	0.027
LVMI (g/m^2^)	86.4±17.1	83.4±17.7	89.1±16.3	0.182
Relative wall thickness	0.34±0.04	0.34±0.03	0.35±0.05	0.285
LAESVI (mL/m^2^)	38.10±9.98	38.32±9.79	37.90±10.30	0.867
End-diastolic volume (mL)	83.8±21.8	75.8±18.8	91.4±21.9	0.003
End-systolic volume (mL)	30.9±12.5	27.0±11.5	34.6±12.6	0.014
Ejection fraction (%)	63.8±5.5	65.0±5.1	62.7±5.7	0.085
LVOTd (cm)	2.11±0.14	2.02±0.10	2.19±0.12	<0.001
Stroke volume (mL)	74.4±15.1	70.0±12.1	78.5±16.5	0.021
Heart rate (beats/min)	68±11	68±9	68±13	0.905
Cardiac output (mL/min)	4,989±935	4,710±771	5,251±1,008	0.019
Cardiac index (mL/min/m^2^)	2,928±540	2,979±549	2,881±537	0.473
E velocity (cm/min)	60.7±16.1	62.4±17.0	59.1±15.3	0.415
E’ velocity (cm/min)	5.98±1.36	6.08±1.56	5.89±1.15	0.569
E/E’ ratio	10.3±2.3	10.4±2.1	10.2±2.6	0.759
LV GLS (%)	-17.86±2.24	-18.65±2.31,	-17.12±1.93	0.005

Values are mean ± standard deviation.

LAESVI left atrial systolic volume index; LV GLS, left ventricular global longitudinal strain; LVIDd, left ventricular internal dimension at diastole; LVIDs, left ventricular internal dimension at systole; LVMI, left ventricular mass index; LVOTd, left ventricular outflow tract diameter

Brachial and central BPs were not different between women and men, but SVRI and AIx75 were greater, and TACI was lower in women. E_LV_I and E_A_I were higher in women, while VAC, Zc, and RM showed no significant differences (Table 3).

**Table 3:** Comparison of hemodynamic data.

	Total (n = 64)	Female (n = 31)	Male (n = 33)	*P* value
Brachial SBP (mmHg)	138.0±13.2	136.6±13.0	139.4±13.5	0.400
Brachial DBP (mmHg)	82.2±9.4	80.2±10.8	84.0±7.5	0.105
SVR (dynes/sec/cm^-5^)	1,702±352	1,760±341	1648±359	0.204
SVRI (dynes/sec/cm^-7^)	1,008±244	1,112±222	911±226	0.001
Central SBP (mmHg)	129.3±13.4	128.3±13.6	130.2±13.4	0.583
Central DBP (mmHg)	83.3±9.6	81.3±11.0	85.2±7.7	0.105
Heart rate (beats/min)	67±11	68±9	67±13	0.629
AIx75 (%)	26.4±8.5	29.3±8.9	23.7±7.1	0.007
TAC (mL/mmHg)	1.39±0.47	1.23±0.34	1.54±0.53	0.006
TACI (mL/mmHg∙m^2^)	0.81±0.23	0.77±0.21	0.84±0.26	0.259
E_LV_ (mmHg/mL)	2.04±0.45	2.11±0.49	1.98±0.42	0.251
E_LV_I (mmHg/mL∙m^2^)	1.21±0.33	1.33±0.34	1.10±0.29	0.004
E_A_ (mmHg/mL)	1.74±0.39	1.81±0.38	1.67±0.39	0.153
E_A_I (mmHg/mL∙m^2^)	1.03±0.28	1.14±0.25	0.93±0.26	0.001
VAC	0.86±0.14	0.88±0.17	0.85±0.11	0.431
Zc (x dyne-sec/cm^3^)	271±124	267±114	275±135	0.801
RM	0.778±0.082	0.775±0.078	0.780±0.086	0.797

Values are mean ± standard deviation.

AIx75, augmentation index corrected at heart rate 75/min; DBP, diastolic blood pressure; E_A_, effective arterial elastance; E_A_I, effective arterial elastance index; E_LV_, left ventricular end-systolic elastance; E_LV_I, left ventricular end-systolic elastance index; RM, reflection magnitude; SBP, systolic blood pressure; SVR, systemic vascular resistance; SVRI, systemic vascular resistance index; TAC, total arterial compliance; TACI, total arterial compliance index; VAC, ventricular arterial coupling; Zc, characteristic impedance

### Associations of E’ velocity and LV GLS with hemodynamic data

**Fig 1:**
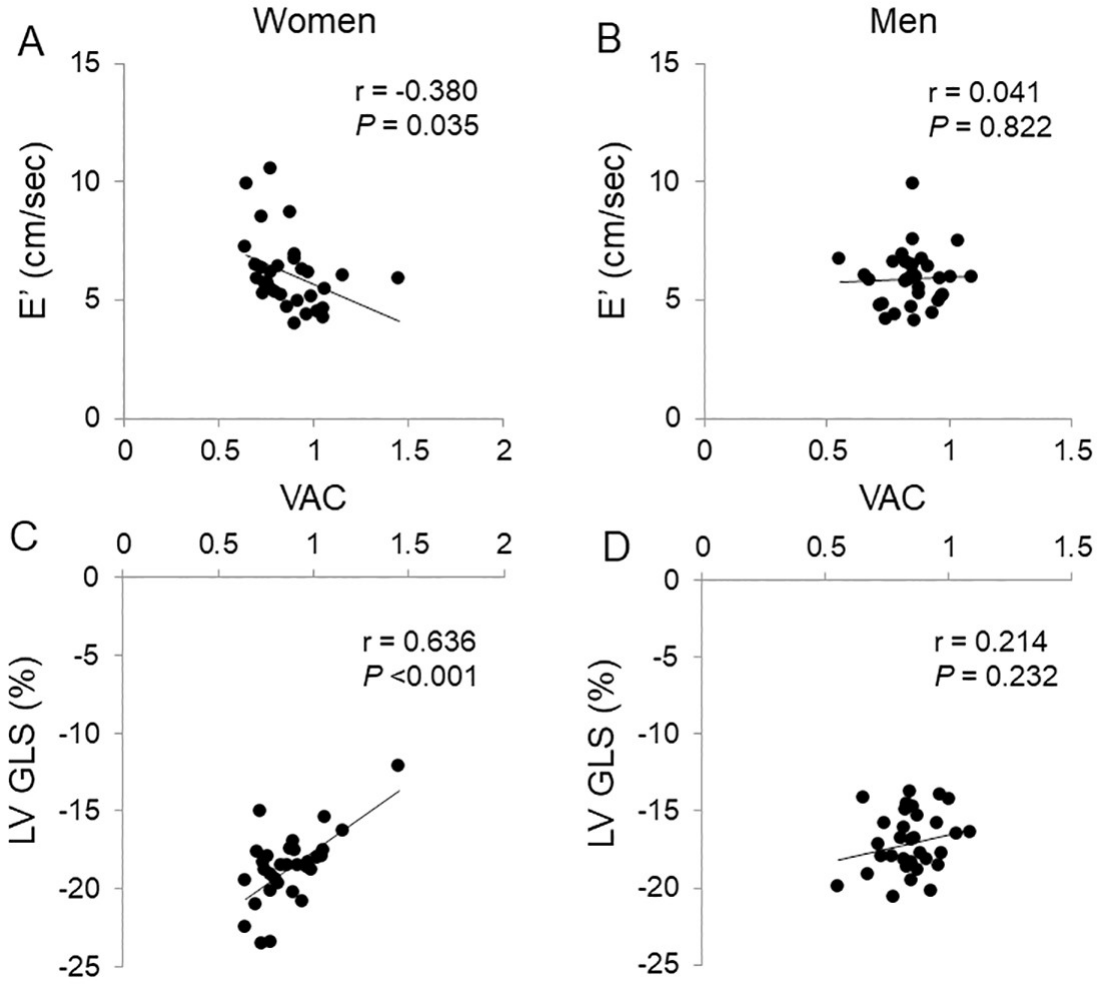
Sex differences in correlations of ventricular-arterial coupling (VAC) with E’ velocity (cm/sec) and left ventricular global longitudinal strain (LV GLS, in %). (A) VAC and E’ velocity in women. (B) VAC and E’ velocity in men. (C) VAC and LV GLS in women. (D) VAC and LV GLS in men.

In women, E’ velocity demonstrated a negative correlation with VAC (Fig 1 and S2 Table). In the linear regression analysis after adjusting for age, height, and central SBP, E’ velocity still exhibited a significant associations with VAC (beta -0.344; *P* = 0.029) (Table 4). However, in men, the association of E’ velocity with VAC was not demonstrated. E_A_I, E_LV_I, Zc, RM, SVRI and TACI showed no associations with E’ velocities in both women and men (S2 Table and Table 4).

**Table 4:** Linear regression analysis of the determinants of E’ velocity.

	Female				Male			
	Unadjusted		Adjusted*		Unadjusted		Adjusted*	
	Beta	*P* value	Beta	*P* value	Beta	*P* value	Beta	*P* value
VAC	-0.380	0.035	-0.344	0.029	0.041	0.227	0.025	0.889
E_A_I	-0.217	0.242	-0.021	0.909	0.302	0.088	0.297	0.129
E_LV_I	0.125	0.504	0.313	0.068	0.332	0.059	0.381	0.063
Zc	-0.010	0.958	0.206	0.246	0.074	0.684	0.261	0.278
RM	-0.098	0.599	-0.152	0.349	-0.072	0.692	-0.295	0.203
SVRI	-0.086	0.646			0.324	0.066		
TACI	0.249	0.176			-0.142	0.430		
Age	-0.529	0.002			-0.341	0.052		
Height	0.152	0.415			0.074	0.683		
CSBP	-0.369	0.041			-0.051	0.779		

*adjusted for age, height, CSBP

CSBP, central systolic blood pressure; E_A_I, effective arterial elastance index; E_LV_I, left ventricular end-systolic elastance index; RM, reflection magnitude; SVRI, systemic vascular resistance index; TACI, total arterial compliance index; VAC, ventricular arterial coupling; Zc, characteristic impedance

Conversely, LV GLS displayed a positive correlation with VAC in women (Fig 1 and S2 Table). In the adjusted linear regression analysis, after accounting for age, height, and LVEF, significant associations of LV GLS with VAC (beta 0.470; *P* = 0.012) and TACI (beta -0.348; *P* = 0.030) were demonstrated (Table 5). However, in men, such an association was not demonstrated. E_A_I, E_LV_I, Zc, RM and SVRI showed no associations with LV GLS in both women and men (S2 Table and Table 5).

**Table 5:** Linear regression analysis of the determinants of left ventricular global longitudinal strain.

	Female				Male			
	Unadjusted		Adjusted*		Unadjusted		Adjusted*	
	Beta	*P* value	Beta	*P* value	Beta	*P* value	Beta	*P* value
VAC	0.636	<0.001	0.470	0.012	0.214	0.232	0.003	0.987
E_A_I	0.328	0.072	0.238	0.161	0.124	0.493	0.057	0.718
E_LV_I	-0.120	0.521	-0.042	0.806	0.011	0.951	0.067	0.697
Zc	0.051	0.783	-0.196	0.200	0.133	0.460	0.149	0.384
RM	0.329	0.071	0.138	0.362	-0.066	0.716	-0.156	0.366
SVRI	0.406	0.023	0.242	0.180	0.153	0.394		
TACI	-0.366	0.043	-0.348	0.030	-0.155	0.388		
Age	0.119	0.523			-0.406	0.019		
Height	-0.005	0.979			0.016	0.929		
EF	-0.528	0.002			-0.648	<0.001		

*adjusted for age, height, EF

E_A_I, effective arterial elastance index; EF, ejection fraction; E_LV_I, left ventricular end-systolic elastance index; RM, reflection magnitude; SVRI, systemic vascular resistance index; TACI, total arterial compliance index; VAC, ventricular arterial coupling; Zc, characteristic impedance

The E/E’ ratio showed no correlation with VAC, E_A_I, E_LV_I, Zc, and RM in women. In men, it showed negative correlations with E_A_I and E_LV_I (S2 and S3 Tables).

### Changes in echocardiographic and hemodynamic data after handgrip exercise

SBP, DBP, HR, and CI increased after handgrip exercise, while SVRI, TACI, EF, E’ velocity and LV GLS showed no significant change in both sexes. In women, compared with men, the E_A_I and E_LV_I were higher than those of men, but E_A_I, E_LV_I, and VAC did not significantly change after handgrip exercise. However, in men, E_LV_I increased, and VAC decreased significantly after handgrip exercise (Table 6). Zc and RM did not change after handgrip exercise in both sexes.

**Table 6:** Changes of echocardiographic and hemodynamic data after handgrip exercise.

	Female (n = 31)			Male (n = 33)		
	Baseline	Exercise	*P* value	Baseline	Exercise	*P* value
Brachial SBP (mmHg)	136.6±13.0	142.9±19.9	0.014	139.4±13.5	144.9±13.2	0.004
Brachial DBP (mmHg)	80.2±10.8	85.2±10.0*	<0.001	84.0±7.5	90.7±10.3	<0.001
Central SBP (mmHg)	128.3±13.6	135.4±20.0	0.003	130.2±13.4	136.2±13.3	0.001
Central DBP (mmHg)	81.3±11.0	86.2±10.2*	0.001	85.2±7.7	91.7±10.4	<0.001
HR (beats/min)	68±9	71±9	<0.001	68±13	71±13	0.001
SV (mL)	70.0±12.1	70.5±10.2	0.649	78.5±16.5	79.8±16.6	0.367
CI (mL/min/m^2^)	2979±549	3138±529	0.013	2881±537	3043±578	0.010
SVRI (dynes/sec/cm^-7^)	1112±222*	1112±214*	0.980	911±226	913±225	0.943
TACI (mL/mmHg∙m^2^)	0.77±0.21*	0.79±0.27*	0.703	0.84±0.26	0.88±0.28	0.274
EF (%)	65.0±5.1	64.7±4.9	0.357	62.7±5.7	62.6±5.7	0.961
E’ velocity (cm/sec)	6.08±1.56	6.34±1.76	0.137	5.89±1.15	6.06±1.36	0.325
LV GLS (%)	-18.65±2.31*	-18.88±2.63*	0.402	-17.12±1.93	-16.91±2.17	0.330
E_A_I (mmHg/mL∙m^2^)	1.14±0.25*	1.18±0.24*	0.198	0.93±0.26	0.95±0.26	0.430
E_LV_I (mmHg/mL∙m^2^)	1.33±0.34*	1.38±0.31*	0.156	1.10±0.29	1.19±0.38	0.021
VAC	0.88±0.17	0.87±0.16	0.349	0.85±0.11	0.81±0.12	0.035
Zc (x dyne-sec/cm^3^)	267±114	269±138	0.897	275±135	287±166	0.509
RM	0.775±0.078	0.772±0.078	0.853	0.780±0.086	0.773±0.100	0.498

Values are mean ± standard deviation.

*P <0.05 vs. male

CI, cardiac index;, DBP, diastolic blood pressure; E_A_I, effective arterial elastance index; E_LV_I, left ventricular end-systolic elastance index; EF, ejection fraction; HR, heart rate; LV GLS, left ventricular global longitudinal strain; RM, reflection magnitude; SBP, systolic blood pressure; SVRI, systemic vascular resistance index; TACI, total arterial compliance index; VAC, ventricular arterial coupling; Zc, characteristic impedance

**Fig 2:**
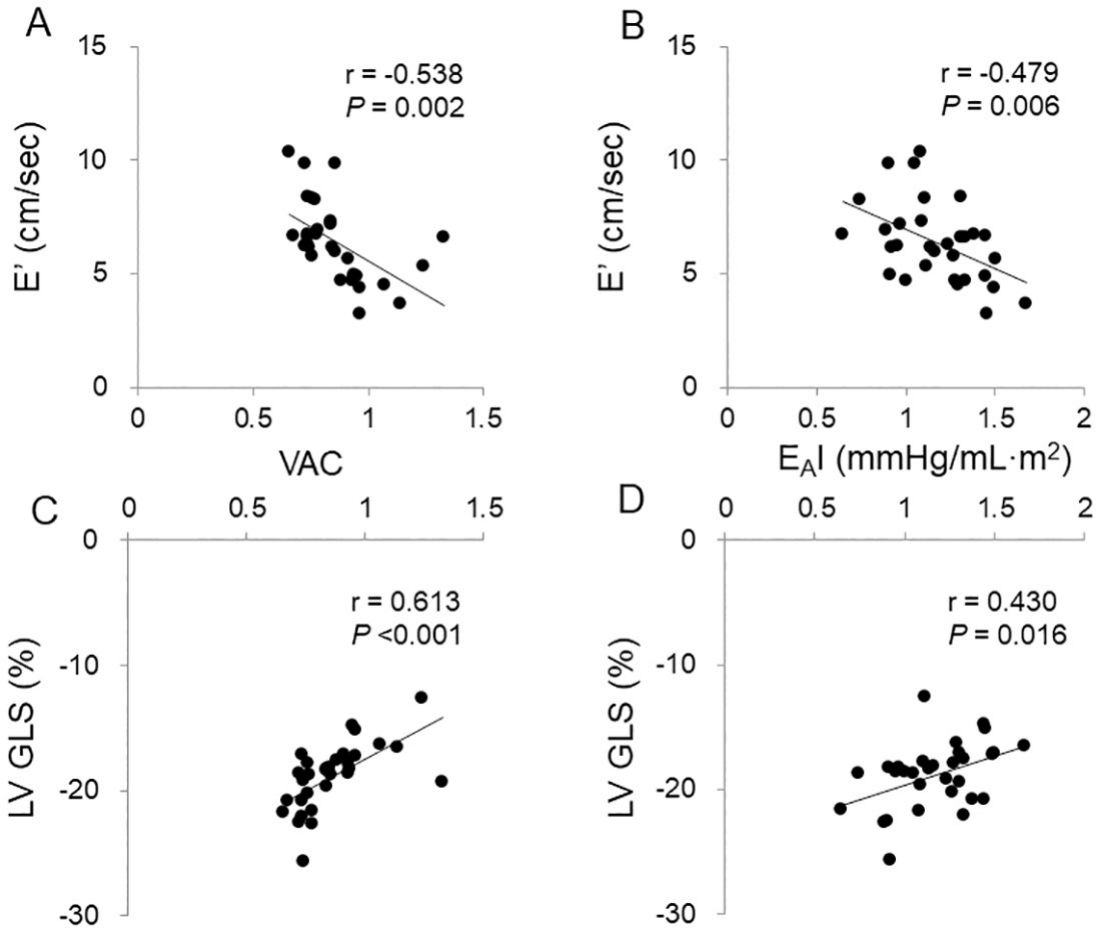
Correlations of ventricular-arterial coupling (VAC) and effective arterial elastance index (E_A_I, in mmHg/ml∙m^2^) with E’ velocity (cm/sec) and left ventricular global longitudinal strain (LV GLS, in %) after handgrip exercise in women. (A) VAC and E’ velocity. (B) E_A_I and E’ velocity. (C) VAC and LV GLS. (D) E_A_I and LV GLS.

After handgrip exercise, VAC exhibited a significant correlation with E’ velocity and LV GLS in women (Fig 2). In the linear regression analysis after adjustment, VAC continued to demonstrate significant associations with E’ velocity (beta -0.488; *P* <0.001) and LV GLS (beta 0.437; *P* = 0.021) in women (S4 and S5 Tables). The E_A_I also showed a significant correlation with E’ velocity and LV GLS after handgrip exercise in women (Fig 2). The association of E_A_I with LV GLS remained significant in the adjusted linear regression analysis, after accounting for age, height, and LVEF (beta 0.359; *P* = 0.047) (S5 Table). Additionally, SVRI (beta 0.396; *P* = 0.035) and TACI (beta -0.479; *P* = 0.010) were significantly associated with LV GLS in women. However, in men, such an association was not demonstrated after handgrip exercise.

## Discussion

The main findings of this study were that hypertensive women had greater E_A_I and E_LV_I than hypertensive men, indicating that hypertensive women have stiffer aorta and LV. Hypertensive women exhibited impaired LV contractile and VAC reserve after handgrip exercise. Additionally, ventricular-arterial mismatch had a notable impact on LV diastolic and systolic dysfunction only in women. These results confirmed the sex differences in the association between VAC and LV dysfunction in response to increased LV afterload. These findings suggest that in hypertensive women without HF, therapeutic considerations are necessary to maintain optimal VAC for the prevention of HFpEF.

Due to the need for invasive catheterization and alterations in LV preload to obtain pressure-volume (PV) loops and end-systolic PV relationship for the collection of VAC and its components, the clinical applicability of VAC was initially limited. Subsequent research, however, demonstrated that E_A_ and E_LV_ could be estimated non-invasively using brachial BP, LVEF, SV and tNd [24–27]. All of these parameters can be easily obtained during the echocardiographic study that was used in this research.

In response to hypertension, women typically exhibit less ventricular dilatation and greater concentric remodeling of LV [10]. However, in our study, despite hypertensive women having smaller LV diastolic and systolic volumes than hypertensive men, relative wall thickness and LV mass index remained comparable due to the smaller LV wall thickness.

After menopause, women were found to have a stiffer arterial system than men [16, 28]. Increased arterial stiffness leads to an elevation in aortic afterload. Both LV diastolic and systolic functions are influenced by aortic afterload, which consists of two components: a steady component and a dynamic component. Micro-vascular characteristic, such as peripheral vascular resistance, is a key determinant of the steady component. On the other hand, the characteristics of conduit arteries, including the proximal aorta’s Zc, the size and position of the augmentation of the reflected wave on the incident wave, and total arterial compliance determine the dynamic component [6, 7].

In this study, women exhibited higher SVRI and AIx75, and lower TACI despite no differences in brachial and central BPs. These findings indicate that women had increased both steady and dynamic aortic afterloads compared with men. Inconsistent results in sex difference of Zc were reported; one study showed higher Zc in women than in men [29], while another study found no sex difference [30]. A recent large population-based study reported higher Zc in women compared to men [31]. However, our study showed no significant sex difference in Zc. As AIx is dependent on various variables beyond the amplitude of the reflected wave, including height, heart rate, and the position of the wave’s arrival, its role as a prognostic factor in clinical events may be limited [5]. In contrast, RM, which necessitates the dissociation of the arterial wave into incident and reflected waves after simultaneous recording of both arterial pressure and flow waveforms, has been reported as a predictor of HF [32, 33]. Women showed higher AIx75, but RM was not different between sexes in this study, which is consistent with a previous finding [30].

In our study, compared with men, women exhibited a higher E_A_I, initially known as a combined index of resistive and pulsatile arterial load [27]. Additionally, E_LV_I was higher in women, while VAC showed no significant difference between genders, consistent with a previous study [10]. However, after handgrip exercise, VAC significantly decreased only in men due to the increase in E_LV_I. Women with hypertension showed no significant changes in VAC and E_LV_I, indicating impaired reserve of LV contraction and VAC in response to handgrip exercise, which is in line with previous researches [15, 34]. Abnormal VAC reserve with exercise was also demonstrated in patients with HFpEF [35].

The diastolic relaxation of the heart can be accurately measured non-invasively, using the E’ velocity of the mitral annulus [36, 37]. A low E’ velocity facilitates the early detection of left ventricular diastolic dysfunction. It has been reported that arterial stiffness, assessed by brachial-ankle PWV, was negatively associated with E’ velocity, specifically in elderly women [17]. Pulse pressure amplification, a central hemodynamic measure of arterial stiffness, demonstrated a positive correlation with E’ velocity, but this association was observed only in women [18]. In our study, only women exhibited a significant association between VAC and E’ velocity at rest and after handgrip exercise, suggesting vulnerability to LV diastolic dysfunction in hypertensive women due to a mismatch between aortic load and LV contraction during exercise. We propose that impaired VAC may be one of the contributing factors to the development of HFpEF in hypertensive women.

LV GLS is reported to be more sensitive than LV EF to detect LV systolic dysfunction from its early stage [38]. In an animal model of HF, VAC was significantly associated with LV GLS [39]. In individuals without cardiovascular diseases, a sex difference in the association between LV GLS and arterial stiffness, estimated using the cardio-ankle vascular index, was demonstrated, with such an association found only in women [40]. Our study revealed that SVRI, TACI and VAC were significantly associated with LV GLS only in women. After handgrip exercise, E_A_I was also associated with LV GLS only in women, suggesting that dynamic and steady aortic afterloads and ventricular-arterial mismatch affect LV systolic function in hypertensive women with preserved EF. Collectively, all these findings suggest that in hypertensive women, optimal VAC is required to maintain LV diastolic and systolic functions and prevent the incident HF. Since antihypertensive therapy has been shown to reduce both arterial and ventricular stiffness, as well as improve VAC and LV function [41], our study suggests that stricter BP control in hypertensive women may help prevent the progression to HFpEF in clinical practice.

## Limitations

This study has several limitations. First, nearly 90% of patients were taking anti-hypertensive medications, which may influence BP, VAC, and its components. However, medications were equally taken between women and men, and baseline clinical characteristics, brachial, and central BPs were not different. Second, a 2-minute handgrip exercise may not provide enough stress to reveal the differences in LV contractile and VAC reserve between genders. However, in our previous study, central systolic and diastolic BPs reached a plateau after a 2-minute handgrip exercise set at 30–40% of maximal handgrip power [21]. Third, sex differences in LV contractile and VAC reserve, as well as the association of VAC with LV functional parameters shown this study may not be solely due to sex differences but rather differences in body size, even though we tried to index hemodynamic parameters by BSA and employed height as one of the adjusting factors in linear regression analysis. Fourth, serum estrogen levels, which are known to affect arterial stiffness [11, 42], were not measured. However, 27 (87%) out of a total of 31 women included in this study were postmenopausal, and none were undergoing hormone replacement therapy. Including more premenopausal women and comparing them with men based on serum sex hormone levels could potentially influence the study results. Fifth, although the estimation of the aortic pressure waveform using radial artery applanation tonometry and a transfer function is widely used in clinical studies, this method has several limitations [43]. Direct acquisition of the aortic pressure waveform via invasive catheterization [21] would provide more accurate results.

## Conclusions

Hypertensive women had greater E_A_I and E_LV_I than hypertensive men, indicating that hypertensive women have stiffer aorta and LV. Hypertensive women exhibited impaired LV contractile and VAC reserve after handgrip exercise. Additionally, ventricular-arterial mismatch had a notable impact on LV diastolic and systolic dysfunction only in women. Therefore, in hypertensive women, optimal VAC is required to maintain LV diastolic and systolic functions and prevent the incident HF. Stricter BP control may be recommended for hypertensive women compared to hypertensive men to prevent the progression to HFpEF.

## Supporting information

S1 Fig An infographic illustrating the study protocol. (A) Measurement of echocardiographic and hemodynamic data. (B) Aortic pressure-flow analysis (C) Measurement repeated after isometric handgrip exercise. AIx75 augmentation index corrected at heart rate 75/min; BP, blood pressure; CO, cardiac output; dPTI, pressure-time index at diastole; E_A_, effective arterial elastance; E_LV_, left ventricular end-systolic elastance; LA, left atrium; LV, left ventricular, LV GLS, left ventricular global longitudinal strain; LVOT, left ventricular outflow tract; PW, pulsed wave; RM reflection magnitude, SV, stroke volume; SVR, systemic vascular resistance; TAC, total arterial compliance; tNd, the ratio of pre-ejection time to total systolic time; VAC, ventricular arterial coupling; Zc, characteristic impedance.
(TIF)

S2 Fig Measurement of tNd, the ratio of the pre-ejection period (PEP) to the total systolic period (pre-ejection period + ejection time [ET]) of ventricular systole. tNd was acquired from the pulsed-wave Doppler tracing of left ventricular outflow tract flow at the apical 5-chamber view as the ratio of the period from ECG Q wave to flow-onset to the period from ECG Q wave to end-flow.
(TIF)

S3 Fig Measurements of aortic characteristic impedance and reflection magnitude. (A) Left ventricular outflow tract flow (LVOT) acquired from pulsed-wave Doppler echocardiography at the apical 5-chamber view. (B) Digitized data of aortic pressure and LVOT flow were aligned to calculated characteristic impedance and reflection magnitude.
(TIF)

S1 Table Lists of the measured and calculated echocardiographic and hemodynamic data. (DOCX)

S2 Table Correlation between indexes of left ventricular diastolic function and ventricular atrial coupling. (DOCX)

S3 Table Linear regression analysis of the determinants of E/E’ ratio. (DOCX)

S4 Table Linear regression analysis of the determinants of E’ velocity after handgrip exercise. (DOCX)

S5 Table Linear regression analysis of the determinants of left ventricular global longitudinal strain after handgrip exercise. (DOCX)

S1 Dataset Minimal raw clinical data with anonymization. (XLSX)
