# Supplementary figures and images for: Sex differences in the impact of ventricular-arterial coupling on left ventricular function in patients with hypertension
Source: PLoS One. 2024 Nov 19;19(11):e0313677. doi: 10.1371/journal.pone.0313677 (PMC11575830; doi:10.1371/journal.pone.0313677)

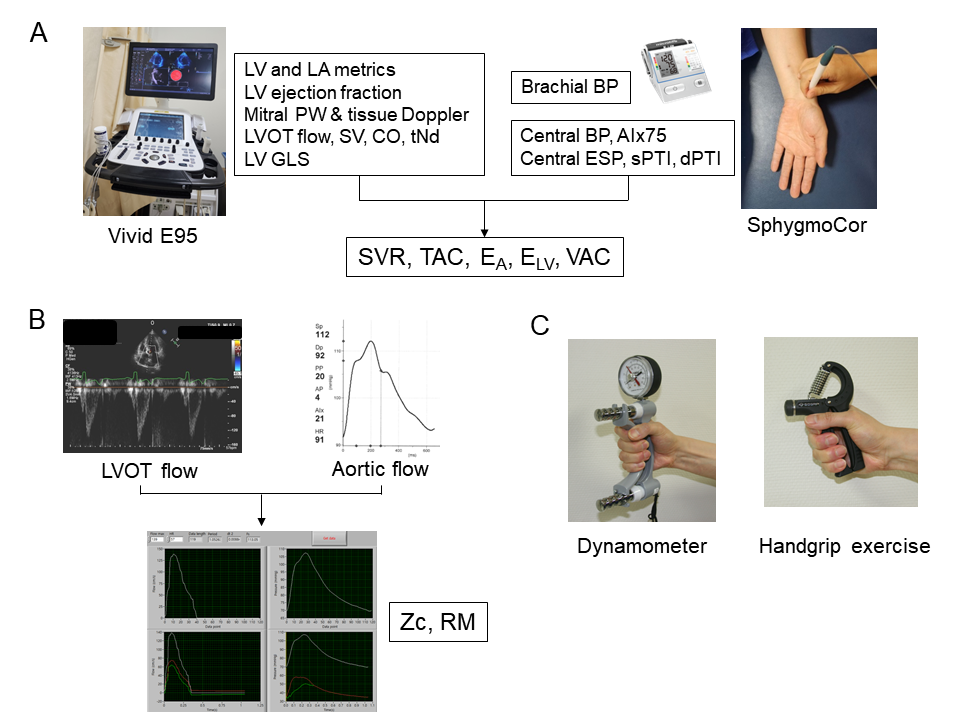

Supplement: S1 Fig — (A) Measurement of echocardiographic and hemodynamic data. (B) Aortic pressure-flow analysis (C) Measurement repeated after isometric handgrip exercise. AIx75 augmentation index corrected at heart rate 75/min; BP, blood pressure; CO, cardiac output; dPTI, pressure-time index at diastole; EA, effective arterial elastance; ELV, left ventricular end-systolic elastance; LA, left atrium; LV, left ventricular, LV GLS, left ventricular global longitudinal strain; LVOT, left ventricular outflow tract; PW, pulsed wave; RM reflection magnitude, SV, stroke volume; SVR, systemic vascular resistance; TAC, total arterial compliance; tNd, the ratio of pre-ejection time to total systolic time; VAC, ventricular arterial coupling; Zc, characteristic impedance. (TIF) [file pone.0313677.s001.tif]

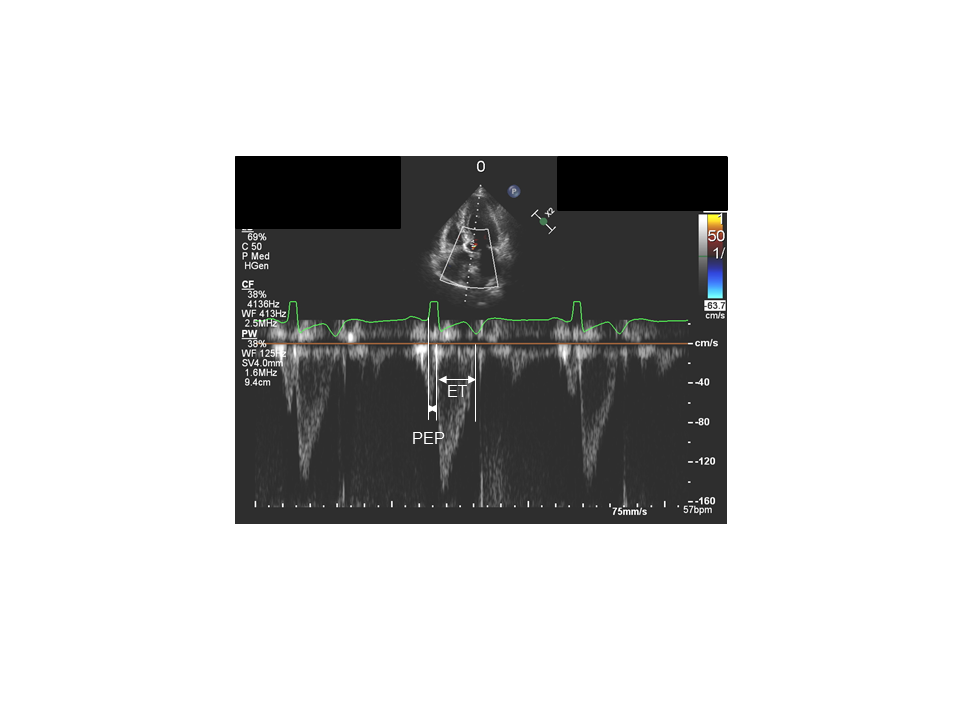

Supplement: S2 Fig — tNd was acquired from the pulsed-wave Doppler tracing of left ventricular outflow tract flow at the apical 5-chamber view as the ratio of the period from ECG Q wave to flow-onset to the period from ECG Q wave to end-flow. (TIF) [file pone.0313677.s002.tif]

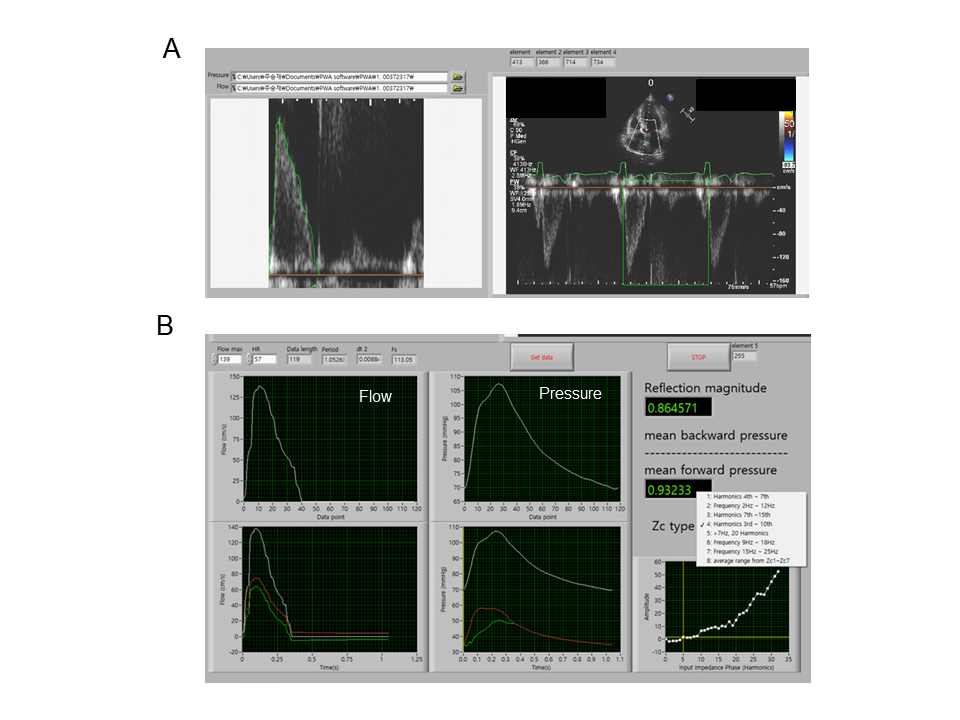

Supplement: S3 Fig — (A) Left ventricular outflow tract flow (LVOT) acquired from pulsed-wave Doppler echocardiography at the apical 5-chamber view. (B) Digitized data of aortic pressure and LVOT flow were aligned to calculated characteristic impedance and reflection magnitude. (TIF) [file pone.0313677.s003.tif]
