# Supplementary material for: Sex differences in the impact of ventricular-arterial coupling on left ventricular function in patients with hypertension
Source: PLoS One. 2024 Nov 19;19(11):e0313677. doi: 10.1371/journal.pone.0313677 (PMC11575830; doi:10.1371/journal.pone.0313677)
Supplement: S1 Table — (DOCX) [file pone.0313677.s004.docx]

S1 Table. Lists of the measured and calculated echocardiographic and hemodynamic data

| **Echocardiographic data** | |
| --- | --- |
| LVIDd, LVIDs, LV wall thickness | From M-mode echocardiography |
| Relative wall thickness | = 2 × LVPWd/LVIDd |
| LV mass | = 0.8 × {1.04 × [([LVIDd + IVSd + LVPWd]^3^ − LVIDd ^3^)]} + 0.6 |
| EDV, ESV, LAESVI | From 2-D echocardiography at apical 4- and 2-chamber views |
| LV ejection fraction | = [(EDV-ESV) /EDV] × 100 |
| LVOT diameter | From 2-D echocardiography at parasternal long-axis view |
| LVOT area | = 3.14 × (LVOT diameter/2)^2^ |
| LVOT flow | TVI from PW Doppler at apical 5-chamber view |
| SV | = LVOT area × LVOT flow |
| CO | = SV × heart rate |
| E velocity | From PW Doppler at apical 4-chamber view |
| E’ velocity | From tissue Doppler at apical 4-chamber view |
| tNd | From LVOT flow waveform at apical 5-chmaber view (Fig S2) |
| LV global longitudinal strain | Using vendor-provided software at apical 2-, 3-, & 4-chamber views |
| **Hemodynamic data** | |
| Brachial SBP & DBP | From digital brachial sphygmomanometer |
| Systemic vascular resistance | = mean brachial BP × 80/CO |
| Central SBP & DBP, AIx75 | From SphygmoCor at the radial artery |
| Central ESP, sPTI, dPTI | From SphygmoCor at the radial artery |
| Total arterial compliance | = (dPTI × SV) / [(sPTI + dPTI) × (central ESP - central DBP)] |
| E_A_ | = brachial SBP × 0.9/SV |
| E_LV_ | Using tNd and single beat approach [24-26] |
| VAC | = E_A_ / E_LV_ |
| Zc, reflection magnitude | From central pressure waveform and LVOT flow (Fig S3) |
| AIx75 augmentation index corrected at heart rate 75/min; CO, cardiac output; D, dimensional; DBP, diastolic blood pressure; dPTI, pressure-time index at diastole; E_A_, effective arterial elastance; E_LV_, left ventricular end-systolic elastance, EDV, end-diastolic volume; ESP, end-systolic pressure; ESV, end-systolic volume; IVSd, interventricular septum thickness at diastole; LAESVI, left atrial systolic volume index; LV, left ventricular, LV GLS, left ventricular global longitudinal strain; LVIDd, left ventricular internal dimension at diastole; LVOT, left ventricular outflow tract; LVPWd, left ventricular posterior wall thickness at diastole; PW, pulsed wave; SBP, systolic blood pressure; sPTI, pressure-time index at systole; SV, stroke volume; tNd, the ratio of pre-ejection time to total systolic time; TVI, time-velocity integral; VAC, ventricular arterial coupling; Zc, characteristic impedance | |
