# Supplementary material for: Sex differences in the impact of ventricular-arterial coupling on left ventricular function in patients with hypertension
Source: PLoS One. 2024 Nov 19;19(11):e0313677. doi: 10.1371/journal.pone.0313677 (PMC11575830; doi:10.1371/journal.pone.0313677)
Supplement: S2 Table — (DOCX) [file pone.0313677.s005.docx]

S2 Table. Correlation between indexes of left ventricular diastolic function and ventricular atrial coupling

|  | All | | Female | | Male | |
| --- | --- | --- | --- | --- | --- | --- |
|  | r | *P* value | r | *P* value | r | *P* value |
| E’ velocity | | | | | | |
| E_A_I | 0.046 | 0.719 | -0.217 | 0.242 | 0.302 | 0.088 |
| E_LV_I | 0.219 | 0.082 | 0.125 | 0.504 | 0.332 | 0.059 |
| VAC | -0.227 | 0.072 | -0.380 | 0.035 | 0.041 | 0.822 |
| Zc | 0.027 | 0.831 | -0.010 | 0.958 | 0.074 | 0.684 |
| RM | -0.086 | 0.498 | -0.098 | 0.599 | -0.072 | 0.692 |
| LV GLS | | | | | | |
| E_A_I | 0.062 | 0.625 | 0.328 | 0.072 | 0.124 | 0.493 |
| E_LV_I | -0.179 | 0.157 | -0.120 | 0.521 | 0.011 | 0.951 |
| VAC | 0.410 | 0.001 | 0.636 | <0.001 | 0.214 | 0.232 |
| Zc | 0.098 | 0.443 | 0.051 | 0.783 | 0.133 | 0.460 |
| RM | 0.135 | 0.286 | 0.329 | 0.071 | -0.066 | 0.716 |
| E/E’ ratio | | | | | | |
| E_A_I | -0.320 | 0.010 | -0.251 | 0.173 | -0.446 | 0.009 |
| E_LV_I | -0.249 | 0.048 | -0.168 | 0.368 | -0.392 | 0.024 |
| VAC | -0.066 | 0.607 | 0.033 | 0.861 | -0.193 | 0.281 |
| Zc | 0.077 | 0.546 | -0.005 | 0.977 | 0.131 | 0.469 |
| RM | -0.144 | 0.255 | -0.049 | 0.792 | -0.207 | 0.248 |
| E_A_I, effective arterial elastance index; E_LV_I, left ventricular end-systolic elastance index; LVGLS, left ventricular global longitudinal strain; RM, reflection magnitude; VAC, ventricular arterial coupling; Zc, characteristic impedance | | | | | | |
