# Supplementary material for: Sex differences in the impact of ventricular-arterial coupling on left ventricular function in patients with hypertension
Source: PLoS One. 2024 Nov 19;19(11):e0313677. doi: 10.1371/journal.pone.0313677 (PMC11575830; doi:10.1371/journal.pone.0313677)
Supplement: S3 Table — (DOCX) [file pone.0313677.s006.docx]

S3 Table. Linear regression analysis of the determinants of E/E’

|  | Female | | | | Male | | | |
| --- | --- | --- | --- | --- | --- | --- | --- | --- |
|  | Unadjusted | | Adjusted* | | Unadjusted | | Adjusted* | |
|  | Beta | *P* value | Beta | *P* value | Beta | *P* value | Beta | *P* value |
| VAC | 0.033 | 0.861 | 0.131 | 0.486 | -0.193 | 0.281 | -0.083 | 0.655 |
| E_A_I | -0.251 | 0.173 | -0.120 | 0.608 | -0.446 | 0.009 | -0.489 | 0.031 |
| E_LV_I | -0.168 | 0.368 | -0.141 | 0.467 | -0.392 | 0.024 | -0.456 | 0.036 |
| Zc | -0.005 | 0.977 | -0.202 | 0.366 | 0.131 | 0.469 | -0.150 | 0.589 |
| RM | -0.049 | 0.792 | 0.272 | 0.176 | -0.207 | 0.248 | 0.186 | 0.551 |
| Age | 0.056 | 0.766 |  |  | 0.147 | 0.415 |  |  |
| Height | -0.090 | 0.631 |  |  | 0.048 | 0.790 |  |  |
| CSBP | 0.141 | 0.448 |  |  | 0.146 | 0.417 |  |  |
| *adjusted for age, height, HR, CPP  CPP, central pulse pressure; E_A_I, effective arterial elastance index; E_LV_I, left ventricular end-systolic elastance index; RM, reflection magnitude; VAC, ventricular arterial coupling; Zc, characteristic impedance | | | | | | | | |
