# Supplementary material for: Sex differences in the impact of ventricular-arterial coupling on left ventricular function in patients with hypertension
Source: PLoS One. 2024 Nov 19;19(11):e0313677. doi: 10.1371/journal.pone.0313677 (PMC11575830; doi:10.1371/journal.pone.0313677)
Supplement: S4 Table — (DOCX) [file pone.0313677.s007.docx]

S4 Table. Linear regression analysis of the determinants of E’ velocity after handgrip exercise

|  | Female | | | | Male | | | |
| --- | --- | --- | --- | --- | --- | --- | --- | --- |
|  | Unadjusted | | Adjusted* | | Unadjusted | | Adjusted* | |
|  | Beta | *P* value | Beta | *P* value | Beta | *P* value | Beta | *P* value |
| VAC | -0.538 | 0.002 | -0.488 | <0.001 | 0.026 | 0.884 | -0.079 | 0.677 |
| E_A_I | -0.479 | 0.006 | -0.222 | 0.242 | 0.054 | 0.764 | 0.317 | 0.162 |
| E_LV_I | 0.017 | 0.926 | 0.407 | 0.019 | 0.019 | 0.917 | 0.295 | 0.168 |
| Zc | -0.328 | 0.072 | -0.114 | 0.515 | -0.081 | 0.653 | 0.219 | 0.317 |
| RM | 0.031 | 0.867 | -0.134 | 0.406 | 0.207 | 0.247 | 0.001 | 0.996 |
| SVRI | -0.398 | 0.027 | -0.075 | 0.702 | -0.009 | 0.962 |  |  |
| TACI | 0.472 | 0.007 | 0.081 | 0.322 | 0.102 | 0.571 |  |  |
| Age | -0.605 | <0.001 |  |  | -0.281 | 0.113 |  |  |
| Height | 0.250 | 0.175 |  |  | 0.300 | 0.090 |  |  |
| CSBP | -0.473 | 0.007 |  |  | -0.215 | 0.230 |  |  |
| *adjusted for age, height, CSBP  CSBP, central systolic blood pressure; E_A_I, effective arterial elastance index; E_LV_I, left ventricular end-systolic elastance index; RM, reflection magnitude; SVRI, systemic vascular resistance index; TACI, total arterial compliance index; VAC, ventricular arterial coupling; Zc, characteristic impedance | | | | | | | | |
