# Supplementary material for: Sex differences in the impact of ventricular-arterial coupling on left ventricular function in patients with hypertension
Source: PLoS One. 2024 Nov 19;19(11):e0313677. doi: 10.1371/journal.pone.0313677 (PMC11575830; doi:10.1371/journal.pone.0313677)
Supplement: S5 Table — (DOCX) [file pone.0313677.s008.docx]

S5 Table. Linear regression analysis of the determinants of left ventricular global longitudinal strain after handgrip exercise

|  | Female | | | | Male | | | |
| --- | --- | --- | --- | --- | --- | --- | --- | --- |
|  | Unadjusted | | Adjusted* | | Unadjusted | | Adjusted* | |
|  | Beta | *P* value | Beta | *P* value | Beta | *P* value | Beta | *P* value |
| VAC | 0.613 | <0.001 | 0.437 | 0.021 | 0.362 | 0.038 | 0.100 | 0.578 |
| E_A_I | 0.430 | 0.016 | 0.359 | 0.047 | 0.186 | 0.301 | 0.019 | 0.921 |
| E_LV_I | -0.085 | 0.648 | -0.012 | 0.946 | -0.060 | 0.741 | -0.051 | 0.788 |
| Zc | 0.425 | 0.017 | 0.305 | 0.070 | 0.107 | 0.553 | 0.053 | 0.772 |
| RM | -0.076 | 0.686 | -0.084 | 0.610 | 0.047 | 0.795 | -0.019 | 0.913 |
| SVRI | 0.490 | 0.005 | 0.396 | 0.035 | 0.268 | 0.132 |  |  |
| TACI | -0.460 | 0.009 | -0.479 | 0.010 | -0.103 | 0.569 |  |  |
| Age | 0.153 | 0.412 |  |  | -0.375 | 0.031 |  |  |
| Height | 0.064 | 0.732 |  |  | -0.077 | 0.669 |  |  |
| EF | -0.537 | 0.002 |  |  | -0.535 | 0.001 |  |  |
| *adjusted for age, height, EF  E_A_I, effective arterial elastance index; EF, ejection fraction; E_LV_I, left ventricular end-systolic elastance index; RM, reflection magnitude; SVRI, systemic vascular resistance index; TACI, total arterial compliance index; VAC, ventricular arterial coupling; Zc, characteristic impedance | | | | | | | | |
